# Does receipt of 5As services have implications for patients’ satisfaction in India?

**DOI:** 10.1186/s12875-014-0209-2

**Published:** 2014-12-17

**Authors:** Divya Persai, Rajmohan Panda, Sudhir Venkatesan, Monika Arora, Jasjit S Ahluwalia

**Affiliations:** Public Health Foundation of India, New Delhi, India; Division of Epidemiology and Public Health, University of Nottingham, Nottingham, UK; Center for Health Equity, University of Minnesota, Minneapolis, MN USA

**Keywords:** Tobacco cessation, 5As interventions, Primary care, India

## Abstract

**Background:**

The 5As model for behavior change counseling is an evidence-based counseling approach. This study aims to explore the relationship between patient satisfaction with counseling services and 5As interventions in tobacco cessation. We also investigated the impact of satisfaction with counseling services on patients’ intention to quit and recommendation of those services to other tobacco users.

**Methods:**

Two cross-sectional surveys were administered among patients and physicians working in primary health care facilities in 12 districts of two states in India. Health facilities and patients were recruited by systematic random and simple random sampling respectively. We limited our analyses to only those patients who were asked about their tobacco consumption. We used multivariable logistic regression to investigate associations between individual components of 5As interventions and patients’ satisfaction with the counseling services.

**Results:**

Patients who reported that they were *‘advised’* to quit (OR: 9.56; 95% CI: 1.89-48.28), ‘*assessed’* for readiness to quit (OR 2.1, 95% CI: 1.07-4.15) and offered cessation *‘assistance’* (OR 2.2, 95% CI: 1.17–4.29) were more satisfied with the counseling services. Patients who were satisfied with the counseling services were five times more likely to have an intention to quit tobacco (OR: 5.45, 95% CI: 3.59 to 8.27) and four times as likely to recommend counseling to other tobacco users (OR 3.83; 95% CI:2.46 -5.96).

**Conclusions:**

Incorporating 5As interventions in the delivery of primary care would likely increase patients’ satisfaction with physicians’ delivered counseling services. Patients’ recommendation of counseling services will aid in demand generation for cessation services in primary care.

## Background

Behavioral counseling interventions in clinical settings are an important means of addressing modifiable risk factors such as lack of physical activity, poor diet and tobacco use [1]. The 5As model i.e. ‘*Ask’, ‘Assess’, ‘Advise’, ‘Assist’ and ‘Arrange’* for tobacco cessation counseling is an evidence-based approach that is feasible for application in primary care [2]. It includes five components: ‘*asking*’ about tobacco use, ‘*advising*’ to quit, ‘*assessing*’ willingness to quit, ‘*assisting*’ the patient in making a quit attempt, and ‘*arranging*’ follow-up to prevent relapse [3].

Health care professionals play an important role in educating their patients about the consequences of tobacco use and in providing counseling for tobacco cessation [4]. In response to strong evidence that advice and assistance from physicians can significantly increase abstinence rates, the Ministry of Health and Family Welfare in India issued tobacco treatment guidelines in 2011 that called on physicians in primary care and other settings to identify and treat every tobacco user seen in a healthcare setting. The guideline specifies that all health care providers must provide 5As counseling as a part of routine health care consultations and dedicated tobacco cessation specialists services should also be set up in health care settings [5]. However, studies in India revealed that patients visiting primary health care facilities neither receive tobacco cessation counseling, nor are referred to tobacco cessation centres [6,7]. Barriers to the counseling process include physicians’ lack of time and relevant knowledge and the failure to understand the chronic nature of tobacco dependence. Physicians also may worry that they will alienate tobacco users and patients may not revisit if counseled in tobacco cessation during clinical visits [8,9]. However, in reality this is not true as studies reveal that smokers who receive tobacco cessation counseling are more, rather than less, satisfied with their physicians’ care [10,11]. It is also well known that one of the factors behind patients’ satisfaction is receipt of counseling services in health care [10,12,13]. There is limited evidence available on patients’ satisfaction with counseling services in relation to tobacco-related care in India and other developing countries. The present study aims to explore in detail the relationship between patient satisfaction with counseling services and individual components of the 5As interventions in tobacco cessation. In the present study we limited our analyses to only those patients who were “*asked”* about their tobacco consumption history by the physicians. We hypothesized that patient who were *“asked”* about tobacco use and later on received ‘*advise’, ‘assess’, ‘assist’ and ‘arrange’* interventions in tobacco cessation would be more satisfied with the counseling services than those who did not receive the 5As cessation services. We also investigated the impact of patients’ satisfaction with counseling services with patients’ intention to quit tobacco and patients’ recommendation of the counseling services to other tobacco users.

## Methods

### Study setting

The data for this study were collected through cross-sectional surveys conducted among patients and physicians in 12 districts of Andhra Pradesh and Gujarat in India in August 2013. The two surveys were administered simultaneously among patients visiting health facilities and physicians working in health facilities providing primary care. Health facilities providing primary care in India include Primary Health Centres (PHC) and Community Health Centres (CHC), which serve as the first point of contact for patients with a healthcare professional in the public sector. Primary healthcare in India is provided by different healthcare providers including doctors trained in medicine and dentistry, and in some cases providers also include practitioners of the indigenous systems of medicine -Ayurveda, Unani, Siddha, and Homeopathic medicine (AYUSH) [14]. The health facilities where the surveys were conducted were selected through systematic random sampling in the selected districts and the study participants were selected through simple random sampling. Patients were approached immediately following the patient-physician interaction. We registered responses from eligible patients (above 18 years of age and current tobacco users) using an interviewer administered questionnaire comprised of five sections– a) Participant eligibility, b) Socio-demographic information, c) Tobacco use information, d) Counseling practices by physicians, and e) Intention to quit tobacco. The questionnaire for physicians’ survey was comprised of four sections – a) Background characteristics, b) Practices in tobacco control c) Knowledge of physicians, d) Attitude towards tobacco control. Self-help material and refreshments were later provided to the patients for their time. The questionnaires were administered by trained interviewers hired from a survey agency.

## Data analysis

### Outcome variables

Patient satisfaction with the counseling services was our main outcome variable assessed using the question “How would you describe your overall satisfaction with the service you received? (Satisfied/Not satisfied)”; it was clarified to the interviewed patients that the ‘services’ referred specifically to tobacco-cessation services. ‘Tobacco-cessation services’ were broadly defined as any attempt by the healthcare provider to address a patient’s tobacco habit, of which the 5As interventions formed components. Since ‘asking’ about a patient’s tobacco consumption history is the first step in any cessation service, we limited our analyses to only those patients who had, at minimum, been in receipt of the ‘Ask’ component of the 5As. Additionally, we had two secondary outcome variables– intention of the patient to quit tobacco (responses were recorded as yes/no) and the likelihood of patients recommending counseling services to others (assessed using the question “Would you recommend other tobacco users to get counseled by doctors for quitting tobacco?” Yes/No).

### Explanatory variables

Common health facilities where both surveys were performed were identified, and individual patients were matched to their physicians through a unique matching code to obtain the two study samples. Socio-demographic factors included from the patients’ survey were age (as a continuous variable), sex, level of education (categorised as ‘uneducated’, ‘less than primary’, ‘primary but less than secondary’ and ‘secondary and above’) and poverty status recorded as above or below the poverty line.

Number of visits to the healthcare facility that the patient made over the past 12 months was also included as covariates in the analyses; this was categorised as ‘1 or 2 times’, ‘3 to 5 times’ and ‘6 times or more’. Variables pertaining to physicians included age (as a continuous variable), sex and qualification. Qualification of the physicians was categorised as– ‘Medical degree’, ‘Dental degree’ and ‘Ayurveda, Unani, Siddha, and Homeopathic medicine (AYUSH) and others’.

We also explored patients’ perspective on the effect of counseling for quitting tobacco use on a 5 point Likert scale (Strongly agree (5), agree (4), neither agree nor disagree (3), disagree, (2), and strongly disagree (1)). The response was captured by the question: Do you feel counseling/advise would help in quitting tobacco use? Duration of tobacco cessation counseling’ was recorded as a categorical variable (‘Less than 30 seconds’, ‘30 seconds to 1 minute’, ‘1 to 5 minutes’ and ‘6 minutes or more’).

### Statistical analysis

The main outcome of interest for the present analysis was patients’ satisfaction with counseling services. We investigated the influence of the 5As interventions in tobacco cessation– ‘*ask’, ‘advise’, ‘assess’, ‘assist’ and ‘arrange’*– on patient reported satisfaction with the counseling services. However, since our outcome variable (patients’ satisfaction with counseling) was dependent on patients receiving counseling, we limited our analyses to only those patients who were asked about their tobacco consumption history (n = 702). Consequently, we only examined the influence of the remaining four components of the 5As– ‘*advise’ patient to quit, ‘assessing willingness to quit’, ‘assisting’* the patient in making a quit attempt by providing self-help material for tobacco cessation and advice on tobacco cessation and ‘*arranging*’ follow-up counseling – on patient satisfaction. Following this, we investigated the impact of satisfaction with counseling on patients’ intention to quit tobacco among the patients who received counseling services. Finally, we used multivariable logistic regression to explore the association between patient satisfaction with counseling and the likelihood of them recommending it to others. All analyses were adjusted for a set of patient-related characteristics: age, sex, poverty status, level of education and number of visits to a healthcare facility in the past twelve months. A set of physicians-related characteristics such as age, sex and qualification were also adjusted in multivariable regression model. Statistical analyses were carried out using Stata 12.0 [15]. The study was approved by the Public Health Foundation of India, Institutional Ethical Committee (IEC 65/60). Informed consent was taken from physicians and verbal consent was sought from patients and they were informed that the survey was a part of a research study designed to assess how physicians addressed patients’ tobacco use.

## Results

A total of 1549 patients were interviewed in the survey. The response rate of the survey was 95%. In our survey we found that less than 50% of the total number of patients had been asked about tobacco consumption history (n = 702). Table 1 displays the socio-demographic characteristics of the 702 respondents who reported being asked about tobacco use at the time of a primary care visit. The median age of the patients was 35 years (range of 28–48 years) and the majority of patients were males (92%). About two-third of the patients lived below the poverty line (72%).

**Table 1:** **Background characteristics of the respondents**

**Background Characteristics (Total = 702)**	**n (%)**
Age, in years (median, IQR)	36 (28 – 48)
Sex	
Female	56 (8)
Male	646 (92)
Education	
No education	181 (25.8)
Less than primary	199 (28.4)
Primary but less than secondary	213 (30.3)
Secondary and above	109 (15.5)
Socio-economic status	
Below poverty line	515 (73.4)
Above poverty line	187 (26.6)
Number of visits to a physician in the past 12 months	
1 or 2 visits	293 (41.7)
3 to 5 visits	343 (48.9)
6 visits or greater	66 (9.4)
Patient reported receipt of:	
Advise	690 (98.3)
Assess	646 (92)
Assist	643 (91.6)
Arrange	516 (73.5)
Satisfaction with tobacco cessation counseling	
Not satisfied	173 (24.6)
Satisfied	529 (75.4)
Intention to quit	
No	226 (32.2)
Yes	476 (67.8)
Recommendation of tobacco cessation services	
No	154 (21.9)
Yes	548 (78.1)

A total of 345 physicians were interviewed. Out of these 345 physicians, there were 142 physicians who attended to the 702 patients. The average number of patients seen by the physicians per day was 80. The median age of the physicians was 35 years (Inter Quartile Range: 29 to 42). About 83% of the patients were attended by physicians with a medical qualification, 8% of the patients were attended by a physician with a dental qualification and 9% of them were attended by a physician with an Ayurveda, Unani, Siddha, and Homeopathic medicine (AYUSH) qualification.

As highlighted in Table 2, among the patients who has been ‘*asked’* about tobacco reported that their physicians had provided the *‘advise’, ‘assess’, ‘assist’ and ‘arrange’* interventions at high rates: 98.3% of tobacco users were ‘*advised*’ for tobacco cessation, 92% were ‘*assessed*’ for their willingness to quit and ‘*assisted*’ with quitting and 73.5% were informed about follow-up counseling. About one-third of the patients’ (35%) ‘Strongly agreed’ with the statement that counseling would help in quitting tobacco use, whereas, about half of the patients (49%) ‘agreed’ with the statement. Less than 1% of the patients ‘strongly disagreed’ with the statement that counseling would help in quitting tobacco use.

**Table 2:** **Impact of ‘Assess’, ‘Advise’, ‘Assist’ and ‘Arrange’ on patients’ satisfaction with counseling services**

	**95% confidence interval**	**P- value**
(n = 702)		
Advise	9.56(1.89 – 48.28)	0.006
Assess	2.10(1.07 – 4.15)	0.032
Assist	2.24(1.17 – 4.29)	0.015
Arrange	1.00(0.64 – 1.57)	0.998
**Background characteristics: patients**		
Age of the patient	1.01(1.00 – 1.03)	0.092
**Sex of the patient**		
Male	Reference	
Female	1.94(1.03 – 3.64)	0.039
**Level of Education**		
No education	Reference	
Less than primary	1.10(0.65 – 1.86)	0.722
Primary but less than secondary	1.96(1.09 – 3.52)	0.024
Secondary and above	3.36(1.53 – 7.37)	0.003
**Socio-economic status**		
Below poverty line	Reference	
Above poverty line	0.74(0.48 – 1.15)	0.182
**Number of visits to the physician in past 12 months**		
1 or 2 visits	reference	
3 to 5 visits	0.97(0.65 – 1.44)	0.864
6 visits or greater	0.72(0.38 – 1.38)	0.321
**Background characteristics: Physicians**		
Physician qualification		
Medical degree	Reference	
Dental degree	0.82(0.38 – 1.76)	0.613
AYUSH or others	0.90(0.47 – 1.73)	0.75
Gender		
Male	Reference	
Female	0.81(0.54 – 1.22)	0.324
Age	1.03(1.01 – 1.05)	0.007

### Socio-Demographic characteristics and patients’ satisfaction with counseling services

Findings indicate that females were about two times more likely to be satisfied with counseling services as compared to males (OR: 1.94; 95% CI: 1.03 – 3.64). Patients’ who were educated at the secondary school level or higher were over three times more likely to be satisfied with counseling services when compared to those with no education (OR: 3.36; 95% CI: 1.53 **–** 7.37). Other patient-related socio-demographic factors such as the age of the patient, socio-economic status of patients, and physician-related characteristics such as medical qualification and physicians’ gender were not related with patient’s satisfaction with counseling services. However, every one year increase in the age of the physician was associated with a 3% increase in likelihood of patient satisfaction (OR: 1.03; 95% CI: 1.01-1.05).

### 5As intervention in tobacco cessation and patients’ satisfaction with counseling services

About three-fourths of the patients (75%) were satisfied with the counseling services. Patients who reported that they were *‘advised’* to quit were almost ten times more likely to be satisfied with counseling services (OR: 9.56; 95% CI: 1.89-48.28) as compared to patients who did not report they were *‘advised’* to quit. Similarly, satisfaction with counseling services was strongly associated with a patient reporting the provider took the steps of *‘assessing’* patients’ readiness to quit tobacco (adjusted OR 2.1, 95% CI: 1.07-4.15) and offering tobacco cessation *‘assistance’* (adjusted OR2.2, 95% CI: 1.17–4.29; p-value: 0.02). However, ‘*arrange’* for follow-up counseling was not associated with patient reported satisfaction with counseling services (Table 2).

### Patients’ satisfaction with counseling services and ‘intention to quit tobacco’

Our findings indicate that about 68% of the patients had an ‘intention to quit tobacco’ (Table 3). Patients who reported to be satisfied with the counseling services were about five times more likely to have an intention to quit tobacco, when compared with those who were not satisfied with the counseling services (OR: 5.45, 95% CI: 3.59 to 8.27).

**Table 3:** **Satisfaction with counseling services and ‘intention to quit’**

	**Intention to quit tobacco**	
(n = 702)	Odds Ratio^†^ (95% CI)	p-value
Satisfaction (No)	Reference	
Satisfaction (Yes)	5.45 (3.59 – 8.27)	<0.001

^†^Adjusted for socio-demographic factors (age, sex, socio-economic status, level of education, number of visits to a physician in the past 12 months) and physician-related factors (age and gender of the physician and medical qualification).

### Impact of satisfaction with counseling on ‘recommendation of tobacco cessation counseling’

Patients who reported to be satisfied with the counseling were found to be almost four times as likely to recommend counseling to other tobacco users (OR of 3.83; 95% CI:2.46 to 5.96). However, no significant association between duration of tobacco cessation counseling and the possibility of recommendation of tobacco cessation counseling to other tobacco users was found in our analysis.[30 seconds to 1 minute- OR: 0.7 (95% CI:0.34 – 1.44; p-value:0.328); 1 to 5 minutes- OR 1.82 (95% CI:0.86 – 3.85; p-value: 0.115); ≥6 minutes- OR:1.86 (95% CI:0.69 – 5.01)].

## Discussion

In India and many developing countries, there is an emphasis on provision of health services with limited emphasis on patients’ satisfaction. Incorporating patient views into the delivery of services offers one way of making health services patient centric [16]. Patients’ satisfaction with the counseling services is an important measure of improving the quality of the provision of health care services. There are no studies which have assessed patients’ satisfaction with counseling services in relation to tobacco cessation services in primary care settings in India. In the present study, we assessed the association between patients’ satisfaction with counseling services and physicians’ delivered 5As interventions in tobacco cessation.

The socio-demographic determinant of patients’ satisfaction with counseling services has been previously reported. A study conducted by Afzal et.al. in India suggests that demographic characteristics such as age, socio-economic status, and education of patient have a statistically significant effect on satisfaction with the quality of counseling services [17]. Consistent with the findings of this study, we found significant associations between patients’ characteristics such as education and gender with satisfaction with counseling services. A study undertaken by Bertakis et. al. [18] suggests that patients of female physicians were more satisfied than those of male physicians. However, in our study we did not find any significant association between physicians’ gender and patients’ satisfaction with counseling services.

Our findings indicate that a majority of patients strongly agreed that tobacco cessation counseling will help them in quitting tobacco. Similar findings were observed in other studies conducted by India and United States in which patients were positive in their attitudes toward delivery of tobacco cessation counseling services [19]. This finding is promising as it establishes the fact that there is a strong demand from tobacco users for tobacco cessation counseling services in India. This is to emphasise that scaling up cessation services would have acceptability amongst tobacco users. This refutes the fact that tobacco users find counselling services to be nagging and do not prefer to be advised about quitting.

Our findings demonstrated that patients who reported receiving the 5As interventions in tobacco cessation during a visit to their primary care physicians were more satisfied with counseing services than patients who did not report receiving the 5As interventions. Similar findings were observed in other studies across the globe [11-13]. Consistent with the study by Conroy et al. [8], our results suggests that patients who received the ‘*advise’, ‘assess’, ‘assist’ and ‘arrange*’ interventions were about twice as likely to be satisfied with counseling services.

The study by Conroy et al. [8] reported that patients’ satisfaction with counseling services increases incrementally with reports of more intensive 5As services. However, our study findings reveals that patients were more satisfied with the ‘*advise*’ compared to the ‘*assess*’, ‘*assist*’ and ‘*arrange*’ components of 5As interventions. Lower satisfaction with specific components of 5As interventions indicate a need for strengthening these components in order to reinforce the satisfaction benefit associated with providing the full range of 5As interventions.

A study by Robbins et al. in 1993 suggested that the amount of time which a physician spends on health education has an important bearing on patient satisfaction [20]. Our findings do not indicate any significant association between duration of tobacco cessation counseling and satisfaction with the counseling services. In our study, the type of intervention is a more important factor that influences patients’ satisfaction with counseling services as compared to the duration of counseling session.

Very similar to the findings of the study by Conroy et.al [8] conducted in United States, our findings also indicate that patients who are satisfied with counseling services have a higher intention to quit tobacco. Our study implies that patients’ satisfaction with counseling services can eventually increase intention to quit among patients visiting health facilities providing primary care.

Consistent to the findings of the study by Cheng et.al. [21], our findings indicate that patients who are satisfied with counseling services were more likely to recommend other tobacco users to get counseled by physicians. This finding is promising as centres providing primary health care in India have wide outreach and satisfaction with counseling and recommendation to other tobacco users will aid in demand generation for cessation services. At present only 9% of tobacco users have used counseling/advise for cessation [6]. This suggests the need to increase counseling services in tobacco cessation.

Our results must be interpreted with several limitations in mind. First, discussing tobacco cessation could simply be a characteristic of physicians who also are more attentive or more comprehensive in their care, and the higher patients’ satisfaction could reflect these qualities [22]. Limitations also include potential biases if patients were more interested in tobacco cessation, and thus more likely to respond, recall, and report counseling interventions. Respondents in our study under-represented women as well as young tobacco users and reflect the characteristics of patients seeking primary care in public health facilities predominantly in rural areas. Finally, the lack of statistical power in our final analyses meant that we could not adjust for the effects of clustering within each of the twelve districts as well as other potential confounding variables pertaining to patients’ tobacco consumption history.

## Conclusions

Despite these limitations, findings of our study are important as it sheds light on patients’ satisfaction with counseling services in primary care settings. Our findings suggest that if physicians provide cessation advice, assess readiness to quit, and offer assistance through counseling, this will not only aid in tobacco cessation but will also increase satisfaction with counseling services. Demonstrating that physician delivered tobacco cessation intervention will increase patients’ satisfaction with counseling services may increase the likelihood of implementation of these interventions by physicians. In the last few years the Non Communicable Diseases Division at the Ministry of health and has been setting up counselling services in some pilot districts in India. Our findings provide good evidence that these services needs to be scaled up even at primary care. Policy makers should also ensure policies focussed on active monitoring of such counseling services in primary care to maintain high levels of patient’s satisfaction. This will also inform policy as it provides a strong argument to invest in tobacco-related counseling services.

## Abbreviations

5As: Ask, Advice, Assess, Assist, Arrange
AYUSH: Ayurveda, Unani, Siddha, and Homeopathic medicine
CHC: Community health centres
PHC: Primary health centre
